# DEPRESSION, FUNCTIONAL DISABILITY, AND ACCESSING HEALTH CARE AMONG OLDER MEN AND WOMEN IN GHANA AND SOUTH AFRICA

**DOI:** 10.1093/geroni/igz038.301

**Published:** 2019-11-08

**Authors:** Candidus C Nwakasi, J Scott Brown

**Affiliations:** Miami University, Oxford, Ohio, United States

## Abstract

Objectives. To inform a preventive approach to mild depression among older Ghanaians and South Africans, this study will investigate the association and possible variabilities between mild depression, functional disability, accessing health care, sociodemographic, and socioeconomic factors across genders in both countries. Methods. Cross-sectional wave 1 (2007-2010) data from WHO’s Study on Global Ageing and Adult Health (SAGE) are used, and a sample of 3871 for Ghana and 3076 for South Africa are analyzed. Binary multiple logistic regression is used to identify the association between mild depression, functional disability status, socioeconomic and sociodemographic factors, and health status. Results. The proportion of mild depression (MD) is 3.78% and 8.15% for older Ghanaian men and women, and 2.29% and 11.91% for South African older men and women, respectively. At 95% CI, increased severity (mild and high levels) of functional disability are associated with increased odds of MD in Ghanaian and South African older men and women. Apart from South African older men, older people in the study who do not receive healthcare when needed have increased odds of MD. Sociodemographic and socioeconomic factors are also associated with MD. Discussion. An untreated, persistent MD may lead to worse conditions with fatal outcomes. Since, mental health care is lacking in both countries, this study recommends policies directed towards support for formal and informal long-term care, and healthcare access to reduce the risks of depression. Thus, this study’s findings may provide relevant information for managing depression among older Ghanaians and South Africans.

